# End-of-life perceptions among physicians in intensive care units managed by anesthesiologists in Germany: a survey about structure, current implementation and deficits

**DOI:** 10.1186/s12871-017-0384-5

**Published:** 2017-07-11

**Authors:** Manfred Weiss, Andrej Michalsen, Anke Toenjes, Franz Porzsolt, Thomas Bein, Marc Theisen, Alexander Brinkmann, Heinrich Groesdonk, Christian Putensen, Friedhelm Bach, Dietrich Henzler, On behalf on the Working Group Epidemiology and Ethics of the German Society of Anesthesiology and Intensive Care Medicine (DGAI)

**Affiliations:** 1 Clinic of Anaesthesiology, University Hospital Medical School, Alber-Einstein-Allee 23, 89081 Ulm, Germany; 2 Department of Anesthesiology and Critical Care Medicine, Tettnang Hospital, Tettnang, Germany; 3 Institute of Clinical Economics, Health Care Research at the Hospital of General and Visceral Surgery University Hospital Ulm, Ulm, Germany; 4 Department of Anaesthesia, University of Regensburg, Regensburg, Germany; 5 Palliative Care Einheit, Anästhesie, operative Intensivmedizin, Schmerztherapie, Raphaelsklinik GmbH, Akademisches Lehrkrankenhaus der Westfälischen Wilhelms-Universität Münster, Münster, Germany; 6 Klinik für Anästhesie, operative Intensivmedizin und spezielle Schmerztherapie, Klinikum Heidenheim, Heidenheim, Germany; 7 Department of Anesthesiology, Intensive Care Medicine and Pain Medicine, Saarland University Medical Center, Homburg/Saar, Germany; 8 Department of Anesthesiology and Intensive Care Medicine, University Hospital Bonn, Bonn, Germany; 9 Klinik für Anästhesiologie, Intensiv-, Transfusions-, Notfallmedizin und Schmerztherapie (AINS), Ev. Krankenhaus Bielefeld, Akad. Lehrkrankenhaus der WWU Münster, Bielefeld, Germany; 10 Universitätsklinik für Anästhesiologie, op. Intensivmedizin, Rettungsmedizin, Schmerztherapie der Ruhr-Universität Bochum, Klinikum Herford, Herford, Germany

**Keywords:** Anesthesiologists, Education, Continuing, End-of-life care, Goals, Intensive care units, Palliative care, Patient care planning, Prognosis, Quality of life, Surveys and questionnaires

## Abstract

**Background:**

Structural aspects and current practice about end-of-life (EOL) decisions in German intensive care units (ICUs) managed by anesthesiologists are unknown. A survey among intensive care anesthesiologists has been conducted to explore current practice, barriers and opinions on EOL decisions in ICU.

**Methods:**

In November 2015, all members of the German Society of Anesthesiology and Intensive Care Medicine (DGAI) and the Association of German Anesthesiologists (BDA) were asked to participate in an online survey to rate the presence or absence and the importance of 50 items. Answers were grouped into three categories considering implementation and relevance: Category 1 reflects high implementation and high relevance, Category 2 low and low, and Category 3 low and high.

**Results:**

Five-hundred and forty-one anesthesiologists responded. Only four items reached ≥90% agreement as being performed “yes, always” or “mostly”, and 29 items were rated “very” or “more important”. A profound discrepancy between current practice and attributed importance was revealed. Twenty-eight items attributed to Category 1, six to Category 2 and sixteen to Category 3. Items characterizing the most urgent need for improvement (Category 3) referred to patient outcome data, preparation of health care directives and interdisciplinary discussion, standard operating procedures, implementation of practical instructions and inclusion of nursing staff and families in the process.

**Conclusion:**

The present survey affirms an urgent need for improvement in EOL practice in German ICUs focusing on advanced care planning, distinct aspects of changing goals of care, implementation of standard operating procedures, continuing education and reporting of outcome data.

**Electronic supplementary material:**

The online version of this article (doi:10.1186/s12871-017-0384-5) contains supplementary material, which is available to authorized users.

## Background

The question of end-of-life (EOL) care has recently reached modern intensive care medicine. Several aspects need to be considered, such as differences in ethnicity, country and medical specialty. An updated summary of published statements on EOL care in the ICU from national societies has currently been presented, highlighting commonalities and differences within and between international regions [1]. Therein, the complexity of EOL care in the ICU within different ethical and cultural environments, particularly relating to withholding and withdrawing life-sustaining treatment while ensuring the alleviation of suffering, has been recognized. Subsequently, the World Federation of Societies of Intensive and Critical Care Medicine has encouraged their member societies to lead the debate and to develop national guidelines and recommendations regarding EOL care in the ICU within each country [1].

At present, data are sparse on the factors associated with EOL decisions in Germany [2]. In Germany, the majority of surgical and interdisciplinary ICUs are managed by anesthesiologists who are also in charge of patient care. Since the status of structural aspects and the relevance of EOL in German ICUs are unknown, we aimed to explore current practice, barriers and opinions on EOL decisions. Due to the increasing importance of EOL-decisions on ICUs and paucity of data, the working group “Epidemiology and Ethics”, a scientific working group with emphasis on ethics within the Scientific Working Group Intensive Care Medicine (WAKI) of the German Society of Anesthesiology and Intensive Care Medicine (DGAI), has been supported by the DGAI who addressed EOL in ICU as one of their strategic goals in 2013. The hypotheses of the present survey were: 1.) EOL standard operating procedures (SOPs) are not regularly used in ICU’s and 2.) There is a discrepancy between the actual (implementation of items) and the desired status (importance of items) on EOL decisions.

## Methods

After the working group had recommended conducting the survey, the methods were discussed in meetings, telephone conferences and emails by the members of the DGAI working group “Epidemiology and Ethics”. The literature about EOL in ICU was researched and with help from an epidemiologist this survey was developed for those items identified as most relevant. Questions were phrased according to publications regarding EOL care and guidelines, such as the Surviving Sepsis Campaign (SSC) 2012 guidelines [3]. After endorsement by the DGAI, 97 questions were reduced to 59 items in order to limit the questionnaire time to 15 min. In the qualitative pilot test, all members of the working group considered the questions relevant, all agreed with the questions, and none of the participants found areas lacking. In November 2015, all members of the DGAI and the Association of German Anesthesiologists (BDA) were invited by email (*n* = 17,044) to participate in the online survey via “www.Umfrageonline.com”. Only one reminder was sent out before closing on December 23rd, 2015.

### Design

The survey included nine items about the structure of the ICU and 50 items about prognostic scores, reporting of individual patient outcomes, goals of care, patient autonomy, standard operating procedures, quality management, limitations of life-sustaining therapy, nursing aspects and concepts of care for dying patients (Table 1). For each item, participants were asked to state current practice, desired status and importance. Current practice was rated on a modified four-point Likert scale as “yes, always”, “often”, “sometimes” or “no, never”. If “sometimes” or “no, never” was marked, participants were asked to rate whether they considered this as a deficiency, and thus, the desired status. Relevance of an item for the respondents in the implementation domains “sometimes” or “no, never” was estimated as: relevance = “deficiency yes” divided by “deficiency yes and no”. Importance was ranked on a four-point Likert-scale ranging from “not important“to “very important”. To reduce complexity and for a clearer presentation, we focused on three subgroups regarding the implementation domain: “always/often”, “sometimes” and “never”, and on two subgroups regarding the importance: “important” and “not important”.

**Table 1:** Questions (Q 1–50) regarding EOL and Categories (C 1–3)

Prognosis and outcome (Q 1–7)
Q1–3 Do you use scores for estimation of prognosis, such as SAPS II or SOFA, to estimate a patient’s individual prognosis? C2
Q1 In general? C2
Q2 With ICU stay <24 h? C2
Q3 With ICU stay >24 h? C2
Q4 Do you receive outcome data regarding long-term survival after hospital discharge? C3
Q5 Do you receive outcome data from patients discharged to other hospitals or rehabilitation centers? C3
Q6 Do you receive outcome data from patients discharged home? C3
Q7 Do you use outcome data from your hospital for your decisions? C3
Goals of care (curative versus palliative) (Q 8–18)
Q8 Do you use principles of palliative care? C1
Q9 Do you address goals of care within 72 h of ICU admission? C1
Q10 Do you discuss goals of care and prognosis with patients and families? C1
Q11 Do you document the items and results of these conversations with patients? C1
Q12 Do you document the items and results of these conversations with relatives? C1
Q13 Do you discuss indications in an interdisciplinary manner? C1
Q14 Do you discuss whether goals are achievable? C1
Q15 Do you discuss ineffective therapy? C1
Q16 Do you establish feasible and realistic treatment goals? C1
Q17 Do you discuss whether a desirable quality of survival is achievable? C1
Q18 Do you decide on and document to allow natural death (AND)? C1
Patient autonomy (Q 19–26)
Q19 Do you document the assumed consent of the patient? C1
Q20 Do you document conversations with relatives regarding the assumed consent of the patient? C1
Q21 Do you document conversations with the patients regarding their priorities regarding their way of life, their perceptions of quality of live, and their wishes for the future? C1
Q22 Do you prepare adequate advanced health care directives (AHDC) which are accepted by all involved parties in case of ICU care and can be applied directly? C3
Q23 Do you have guidelines for dealing with delicate wishes of patients? C3
Q24 Do you have an ethics committee? C1
Q25 Do you perform ethics councils? C3
Q26 Do you perform interdisciplinary ethics case reviews? C3
Standard operating procedures (SOPs), quality management (Q 27–29)
Q27 Do you have SOPs for psychosocial problems? C3
Q28 Do you have SOPs for spiritual problems? C3
Q29 Do you have a room for taking farewell? C1
Which changes in goals of care do you execute in these instances? (Q 30–37)
Q30–31 In case of further deterioration of defined organ functions in patients with advanced severe underlying disease or relevant functional impairments with primarily equal treatment goals of a potentially reversible acute process (i.e., treatment of pneumonia, pulmonary embolism, mass reduction surgery of tumor), do you perform:
Q30 Continuation and escalation of therapy with all consecutive life-sustaining activities? C1
Q31 Change in goals of care, adjustment of therapy to the new goals, usually by limitations of care? C1
Q32 DNR (Do Not Resuscitate) C1
Q33 DNE (Do Not Escalate) C1
Q34 RID (Re-evaluate Indication and De-escalate) C3
Q35 CTC (Comfort Terminal Care) C3
Q36 Is the decision to changing goals of care authorized by a physician, communicated during handover of duty, checked daily and documented in the patient chart / patient data management system? C1
Q37 Do you have a checklist” items for intensive care medicine for individual changes in treatment goals”? C3
Nursing aspects (Q 38–40)
Q38 Do you integrate nurses’ opinions? C1
Q39 Do you implement palliative care concepts, such as adaption of oral care, noise, light, basal stimulation? C1
Q40 Is the nursing staff educated in palliative care? C3
Concepts of care in the terminal phase (Q 40–50)
Q41 Do you use SOPs for EOL? C3
Q42 Do you do an appraisal of the initial situation? C1
Q43 Is there care for others, such as relatives or the primary care physician, once the patient has died? C3
Q44 Do you use the Liverpool pathway of care? C2
Q45 Do you administer diaries of patients? C2
Q46 Do you administer diaries of relatives? C2
Q47 Do you involve relatives to attend when death occurs? C1
Q48 Do you offer attendance by psychologists, social workers, spiritual care? C1
Q49 Do you consider intercultural aspects? C1
Q50 Are visiting hours handled flexible according to the needs of the relatives? C1

Sufficient Category 1 reflects high implementation and high relevance, inessential Category 2 low and low, and unsatisfactory Category 3 low and high, respectively

Considering implementation, relevance and resulting implication, we focused on three subgroups:

**Table:** 

	implementation	relevance	implication
Category 1:	high	high	sufficient
Category 2:	low	low	inessential
Category 3:	low	high	unsatisfactory

## Results

Eight-hundred twenty-one anesthesiologists (4.8% of 17,044 DGAI or BDA member email adresses) responded. The mailing list classified 870 heads of anesthesiology departments. There are no data available how many of these departments run ICUs. After the first email reminder, there was only minimal increase in responses. Thus, no further reminders were sent out. Only completed surveys (*n* = 541) were included in the analysis. Out of the 541 responders, 417 stated the name of their department, resulting in 305 reponders from different departments. Thus, the response rate reflects more than 1/3 of anesthesiology departments in Germany. Almost all questions were answered by the participants, thus, demonstrating the ability of the questionnaire to distinguish between different respondents.

### Structural items

There was a higher likelihood of physicians working in university hospitals to participate in the survey: 19% of respondents, but only 8% of the ICU’s involved were from university hospitals. Thirty-two percent of respondents worked in maximal care hospitals (level 1), 29% in priority care hospitals (level 2), and 39% in basic and regular care hospitals (level 3). Predominantly, experienced physicians participated in this inquiry: 12% were department heads, 8% ICU directors, 32% anesthesia and intensive care specialists and 16% residents. The participants had experience in intensive care medicine less than 5 years in 26%, between 5 and 10 years in 26% and more than 10 years in 48%.

Regarding the institutions the respondents worked at, 53% were public or community hospitals, 17% were privately owned, 24% pertained to churches and 6% to common welfare organizations. The characteristics of the involved ICU’s are presented in Table 2. Since multiple responses were possible regarding treated patients, the sum of treated patients exceeds 100%.

**Table 2:** Characteristics of involved ICU’s from 541 responses

ICU type	Surgical	Mixed	Medical				
(%)	31	68	1				
Treated patients	Cardiac surgery	Cardiology	Neurosurgery	Neurology	Trauma		
(%)	7	5	14	7	23		
Treated patients	Otorhinolaryngology	Gynecology	Urology	Pediatrics	Pediatric surgery		
(%)	9	17	15	1	2		
Number of ICU beds^2^	≤ 5	<10	11–15	16–20	21–30	31–40	>40
(%)	1	26	28	21	13	6	5
Number of ventilator beds^2^	≤ 5	6–10	11–15	16–20	21–30	31–40	>40
(%)	16	30	24	14	7	4	5
Patients annually	≤ 500	501–1000	1001–1500	1501–2000	> 2000		
(%)	12	30	23	15	21		

### Current practice and estimation of relevance

Only four items reached ≥90% agreement as being performed “yes, always” or “mostly” (questions (Q) Q10, Q12, Q20, Q50). Twenty-nine items were rated by ≥90% of respondents as “very” or “more important”. There was a profound discrepancy between current practice and importance (Table 3, Figs. 1, 2 and 3, Additional files 1, 2, 3, 4, 5, 6, 7, 8, 9 and 10: Figures S1–S10).

**Table 3:** Differentiation of current practice, importance, implementation discrepancy and relevance according to categories

Category	Status of implementation (always / often)	Importance (important)	Implementation discrepancy	Relevance
	(mean ± SD)	(mean ± SD)	(mean ± SD)	(mean ± SD)
Category 1 (%) *n* = 28	71 ± 14	95 ± 4	24 ± 13	75 ± 16
Category 2 (%) *n* = 6	21 ± 23	38 ± 9	17 ± 19	30 ± 7
Category 3 (%) *n* = 16	17 ± 15	79 ± 14	62 ± 15	66 ± 15

Percentage of responses in regards to: status of implementation (always / often), importance (important), implementation discrepancy (difference between percentage values regarding “importance” and “status of implementation always / often”) and relevance (“deficiency yes” divided by “deficiency yes and no” in the implementation domains “sometimes” or “no, never”)

**Fig. 1:**
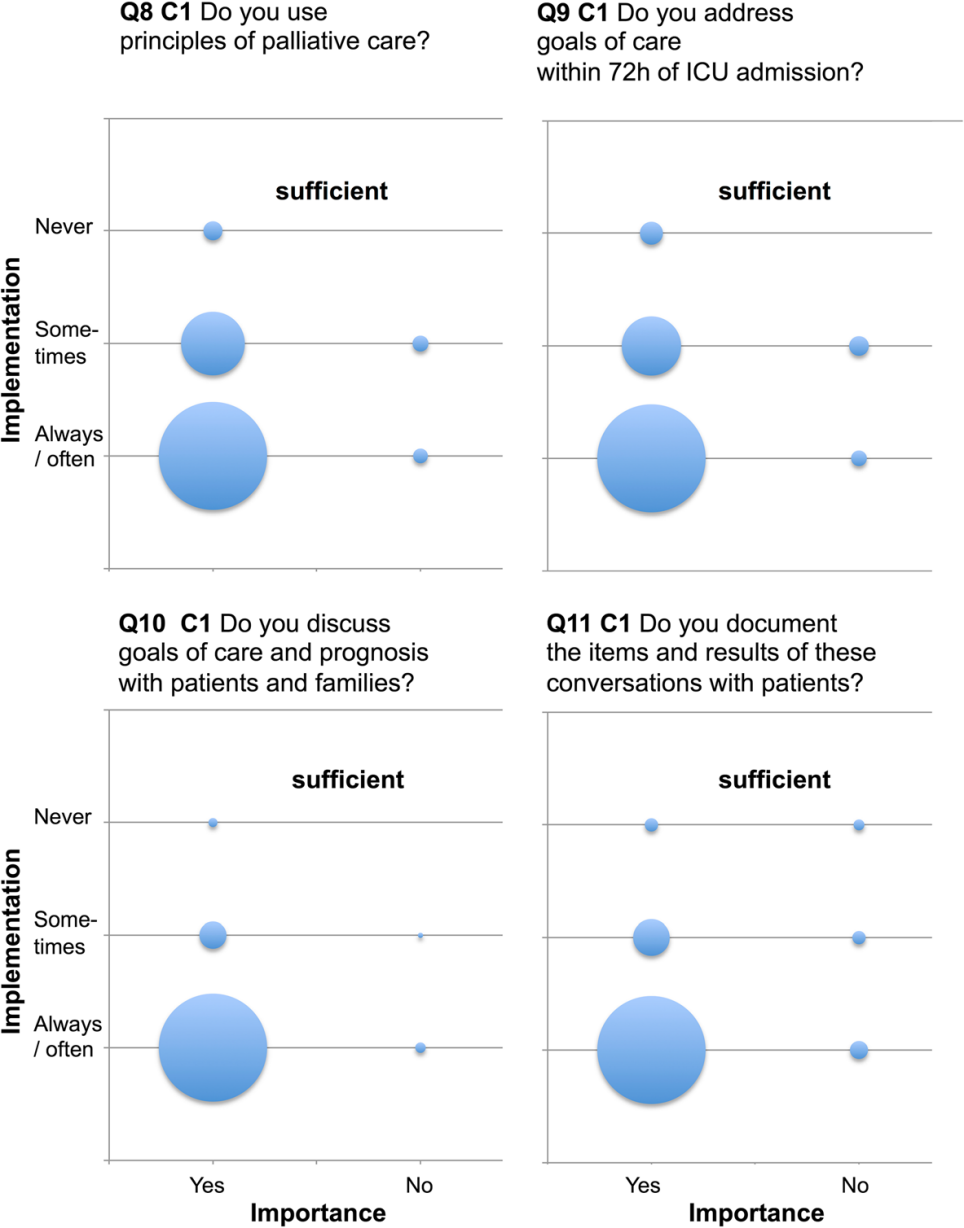
EOL items Q8–11 of high implementation and high relevance (sufficient Category 1). Data are presented as “blob-o-grams” were the number of respondents in each category is represented by a circle whose area is proportional to the number. Importance (x-axis) and status of implementation (y-axis) are rated on modified Likert scales. Q = Question. C1 = sufficient Category 1

**Fig. 2:**
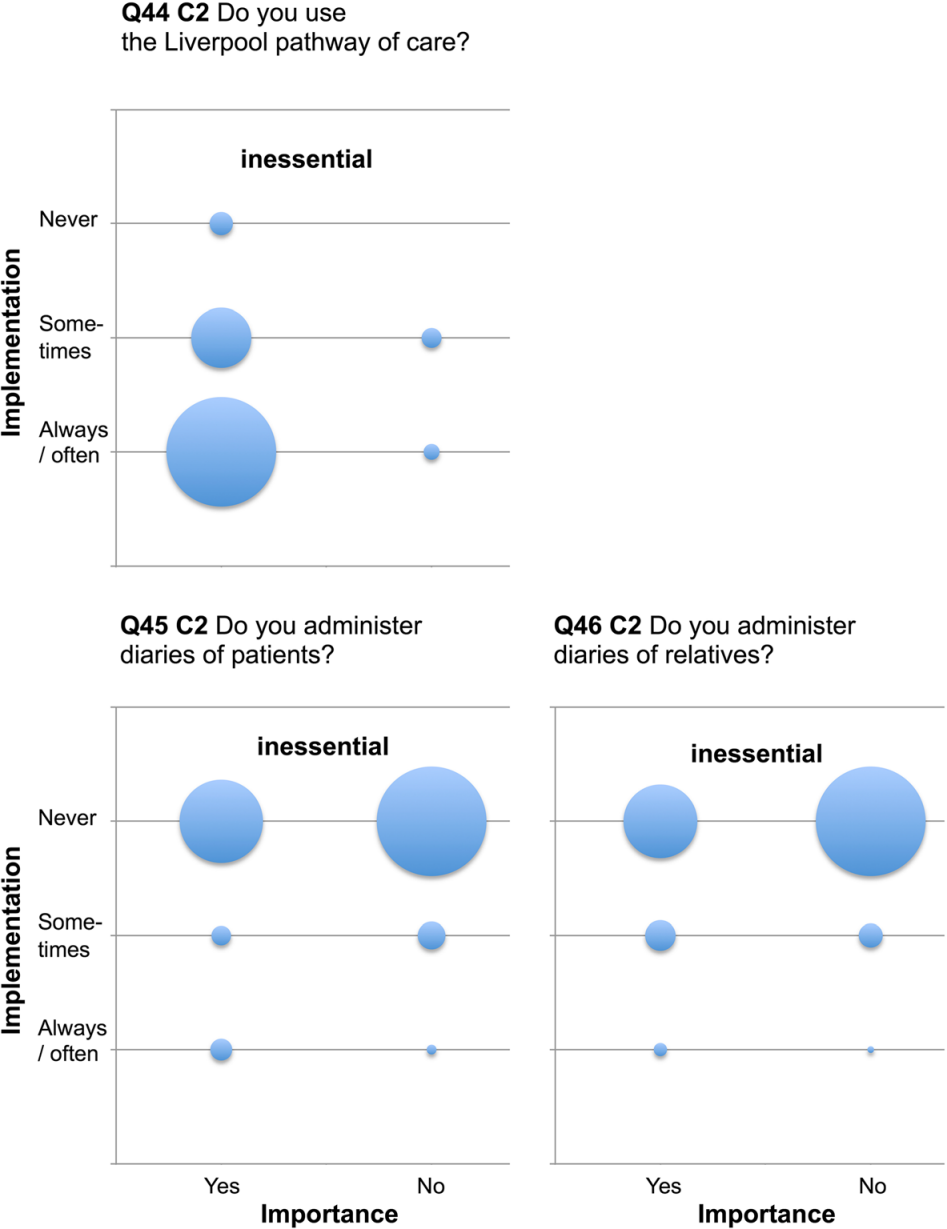
EOL items Q44–46 of low implementation and low relevance (inessential Category 2). Data are presented as “blob-o-grams” were the number of respondents in each category is represented by a circle whose area is proportional to the number. Importance (x-axis) and status of implementation (y-axis) are rated on modified Likert scales. Q = Question. C2 = inessential Category 2

**Fig. 3:**
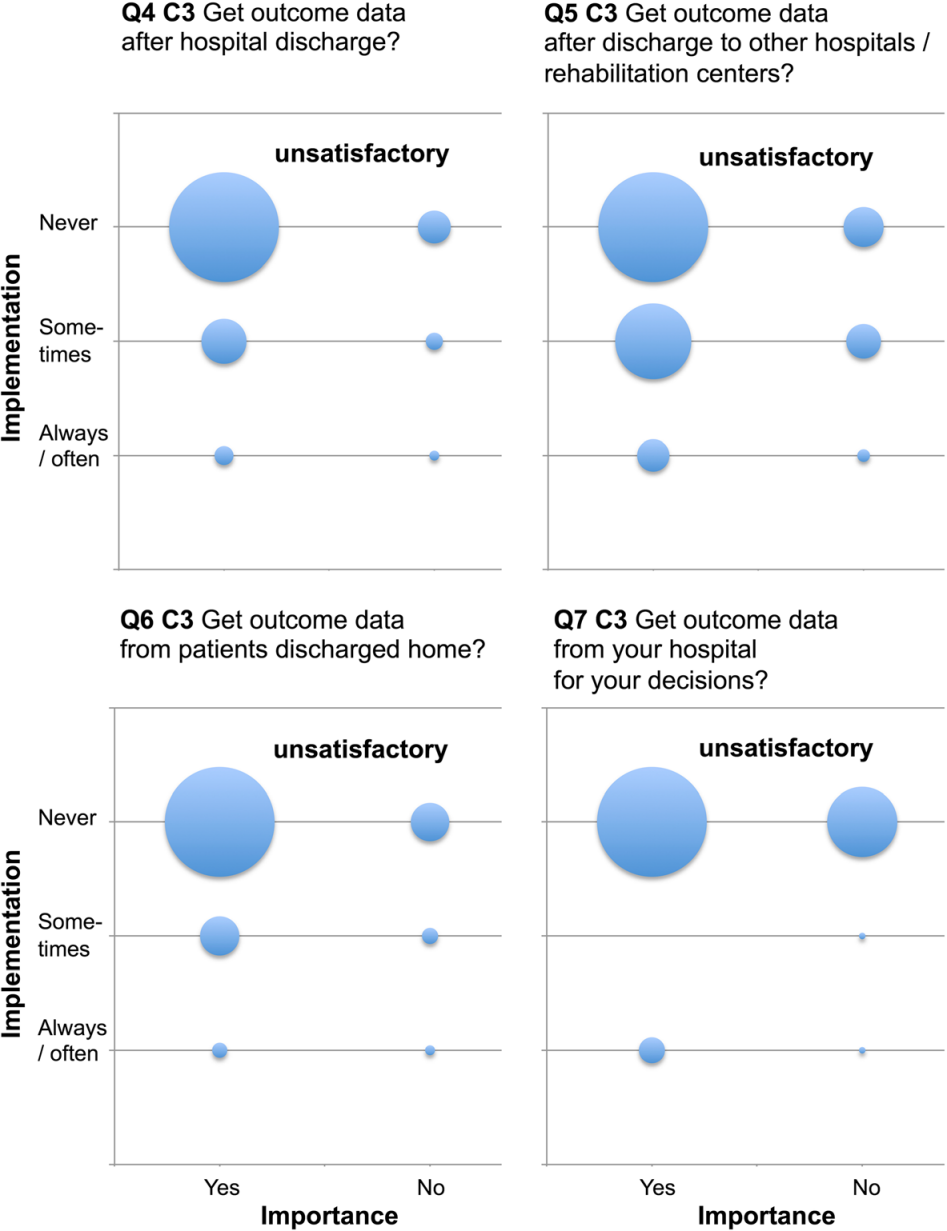
EOL items Q4 – Q7 of high importance that are rarely implemented but are considered to be highly relevant (unsatisfactory Category 3). Data are presented as “blob-o-grams” were the number of respondents in each category is represented by a circle whose area is proportional to the number. Importance (x-axis) and status of implementation (y-axis) are rated on modified Likert scales. Q = Question. C3 = unsatisfactory Category 3

Category 1 (sufficient): Items were of high importance with a high degree of implementation that were judged as relevant by those who didn’t have them (*n* = 28). (Items, Q 8–21, 24, 29–33, 36, 38, 39, 42, 47–50, Fig. 1, Additional files 1, 2, 3, 4, 5 and 6: Figures S1–S6).

Category 2 (inessential): Items were of minor importance with a lesser degree of implementation that were not considered a deficiency by those who didn’t have them (*n* = 6). (Items, Q 1–3, 44–46, Fig. 2, Additional file 7: Figure S7).

Category 3 (unsatisfactory): Items were of high importance that were rarely implemented but were considered to be highly relevant (*n* = 16). (Items, Q 4–7, 22, 23, 25–28, 34, 35, 37, 40, 41, 43, Fig. 3, Additional files 8, 9 and 10: Figures S8–S10).

Of 44 items considered to be important, 24 were attributed to sufficient Category 1 (Table 3, Fig. 1, Additional files 1, 2, 3, 4, 5 and 6: Figures S1–S6) and six to unsatisfactory Category 3 (Table 3, Fig. 3, Additional files 8, 9 and 10: Figures S8–S10). Items were marked as “very” or “more important” by >90% of respondents.

Mean percentages of responses in the three categories are presented in Table 3 regarding implementation (always / often), importance (important), implementation discrepancy (difference between percentage values regarding “importance” and “status of implementation”) and relevance (“deficiency yes” divided by “deficiency yes and no” in the implementation domains “sometimes” or “no, never”). The highest level of importance was found for sufficient Category 1 items which lacked implementation in ¼ of respondents. Unsatisfactory Category 3 items were ranked less important than sufficient Category 1 items but still reached high importance levels. There was a substantial implementation discrepancy characterizing those items with the greatest need for a change in practice, i.e., unsatisfactory Category 3 items. The relevance was profoundly lower than the importance in all categories.

The unsatisfactory Category 3 items characterizing the most urgent need for improvement referred to patient outcome data: Q 4–7 (Fig. 3), preparation of health care directives and interdisciplinary discussion: Q 22, 23, 25, 26 (Additional file 8: Figure S8), development of SOPs: Q 27, 28, the implementation of practical instructions: Q 34, 35, 37 (Additional files 9 and 10: Figures S9 and S10) and the inclusion of nursing staff and families in the process: Q 40, 41, 43 (Additional file 10: Figure S10).

## Discussion

The present survey revealed a discrepancy between current practice of EOL and perceived importance and lack of particular feedback, education, and tools. This was especially important for outcome data and advanced care planning. For the first time, in greater than 1/3 of anesthesiology departments running ICUs in Germany, a survey revealed a valuable insight in current practice, barriers, perceived importance, relevance and deficits of EOL decisions in surgical and interdisciplinary ICUs, which may serve for improvement in EOL. Perhaps, just the most engaged anesthesiologists in EOL have responded because many anesthesiologists do not work in critical care. Thus, their response highlights very informatively where to focus to reduce deficits in German ICUs.

First of all, we wanted to know whether prognostic scores play a role in EOL decisions in German ICUs. SAPS II is regularly recorded on a daily basis in Germany for calculation of diagnosis related groups (DRG’s) and reimbursement. Therefore, we expected that SAPS II might be used for estimation of prognosis, also, which might have influence on EOL decision making. The results of the present survey do not corroborate this hypothesis. Although SAPS II [4] (Simplified Acute Physiology Score) or SOFA [5] (Sequential Organ Failure Assessment) scores were originally developed for estimation of prognosis, they were hardly used for this purpose in the present survey and categorized as inessential Category 2 (Additional file 7 : Figure S7). The prognostic performance of SAPS II is poor [6], better with SAPS 3 [7] and SOFA [5], all predict outcome for a given subgroup of patients, but fail for a single patient. Due to limited ICU resources, physicians seek prediction tools to facilitate allocation of ICU beds to patients which might benefit best. In this context, an initial refusal and final triage score provided objective data for rejecting patients that will die even if admitted to the ICU and survive if refused [8]. The mortality benefit regarding ICU admission appeared greater for the elderly [9].

In absence of adequate prediction models of outcome, the majority of respondents judged feedback on outcome data as very important for individual decision making (Q 4–7, attributed to unsatisfactory Category 3, Fig. 3 ). A considerable variability between hospitals and physicians in terms of EOL care in ICUs is due, in large part, to the lack of compelling evidence or professional consensus for specific approaches that ensure patients of receiving the care they would want if fully informed about their prognosis and likely outcomes [10]. EOL decisions might be improved by establishing interdisciplinary rounds, advanced health care planning, and structured feedback on outcome data.

Questions Q8–18 regarding curative vs. palliative goals of care were answered to be in the sufficient Category 1 (Fig. 1, Additional files 1 and 2: Figures S1 and S2). However, the answers to these goals of care bear a high risk of societally acceptable responses. The issue of “setting goals of care”(Q9 and Q10) has been addressed by the Surviving Sepsis Campaign (SSC) 2012 guidelines [3] and reiterated in the 2016 guidelines [11]. Initiatives to enhance care in the ICU highlight the importance of incorporating goals of care together with the prognosis into treatment plans [12]. It has been reported that less than half of the ICU physicians felt comfortable in having EOL discussions with patients’ families [13]. Therefore, even well-intended clinicians may miss valuable opportunities to address and clarify families’ misunderstandings and concerns regarding goals of care at EOL [14]. Moreover, while the estimations of risks and prognosis may change during hospital stay, patients and families often are unable to move beyond the very first prognostic statements. We emphasize that these skills regarding curative vs. palliative goals of care should be regarded as essential and trained accordingly.

The participants identified SOPs dealing with psychosocial (Q27) and spiritual (Q28) problems as an issue of unsatisfactory Category 3 (Additional file 9: Figure S9). In Germany, an approach to these aspects has been published [15], specifying ethical principles, legal basics, patient autonomy, decision making and implementation regarding limitation of care and change in goals of care, unity of patient and family, cultural and religious influences as well as conflicts and burnout in the caregiving team. In Canada, a series of guidelines address withdrawal, distress and discontinuation [16]. There is a critical need to reframe EOL care planning, not prioritizing life extension over good death [17]. Thus, tools are already available to enable a shift from unsatisfactory Category 3 to sufficient Category 1.

Another deficiency identified was the lack of support regarding “changes in goals of care”(Q34, 35, 37), RID (Re-evaluate Indication and De-escalate) and CTC (Comfort Terminal Care) (Additional files 9 and 10: Figures S9 and S10). Checklists may be helpful, and guidelines for changes in goals of care [18] utilizing DNR (Do Not Resuscitate), DNE (Do Not Escalate), RID and CTC have recently been published, reevaluating, documenting and changing on demand goals of care on a daily basis. However, although acuity of illness and organ dysfunction consistently predicted mortality in critically ill patient populations, only elements of the past medical history were positively associated with a DNR order [19]. The WELPICUS study has achieved world-wide consensus on key EOL issues and terminology [20]. However, EOL decisions are very variable between regions, countries, individual ICU’s and individual clinicians in the same ICU [21, 22], often driven by the views of individual physicians and hospital norms [10]. Instead of “no escalation of treatment”, a “time-limited trial” of life support has been advocated [23]. In practice, withholding preceded or accompanied withdrawal in >90% of patients [22], and was more likely to occur during on-call hours [24]. It is noteworthy that withholding and withdrawal reflect the limitation of life sustaining treatments, but it is essential in the communication with the team, the patient and the relatives that a change in goals of care does not mean the cessation of medical care [25].

Another area of need in the present survey was EOL education (Q40, Additional file 10: Figure S10). The World Health Organization defines palliative care as ‘an approach that improves the quality of life of patients and their families’ [26]. In everyday practice, adoption of the ‘ABCDs’ of EOL critical care is applicable: Attitudes, Behaviours, Compassion and Dialogue [27]. Presently, between 10% and 20% of the population at large now die in the ICU underlining the importance of EOL care to everyday practice and training [25], being extended to EOL orders (Q41) for continuing care after death for relatives (Q43) (Additional file 10: Figure S10). Many clinicians and families equate withholding or withdrawing as giving up [17]. Communication and intervention withdrawal practice guidelines that highlight EOL care as part of, rather than separate from, critical care and education [28] are available and may be crucial in supporting ICU teams to help make good death more accessible [17].

Unfortunately, only a minority of DGAI and BDA members participated in the survey. Thus, the results are not representative. Moreover, due to the voluntary participation, it must be appropriately considered that the most unsatisfied colleagues may have responded willingly, or just the most engaged in EOL. Many anesthesiologists do not work in ICUs and hospitals. Thus, we cannot state how many respondents would be eligible and representative for an EOL survey. It remains unclear, whether EOL is regarded not important or not as a problem. Also, due to the voluntary participation, the distribution of health care providers of respondents was not a representative selection. In 2015, in Germany, 38% of hospitals running ICUs were allocated to public or community hospitals (vs. 53% in present survey), 21% (vs. 17%) to privately owned, and 41% (vs. 30%) to churches and to common welfare organizations [29]. 1177 out of 1956 hospitals stated ICU beds, and 416 intensive care medicine departments [29]. No representative data were available regarding the percentage of hospitals and ICUs in Germany treating **≤** 500 up to >2000 patients / year as given in Table 2. To our surprise, some colleagues reported that they were not permitted to participate by their hospital chief executive officers (CEO’s) for the fear of disclosing sensitive information. Physicians informed their CEO’s and data security engineers, and some were not allowed to participate due to safety concerns with the online survey via “www.Umfrageonline.com”, because this evaluation might not be anonymised as confirmed. This barrier to scientific investigations driven by economic competition is worrisome and could likely increase in the future.

The present survey was only addressed to anesthesiologists, and therefore, the results cannot be generalized to other specialties, professions, or persons affected by EOL decisions, such as nurses, palliative care experts, or families. It is appreciated that surveys regarding EOL care might show different results for different participating subpopulations [10, 21, 22].

## Conclusions

The present survey reveals an urgent need for improvement in EOL practice in German ICUs. Improvement might be achieved by focusing on desirable quality of life, advanced care planning, continuing EOL education and feedback on outcome data. A shift from unsatisfactory Category 3 to sufficient Category 1 may be enabled by generating awareness regarding deficits in EOL care and deliver already available tools via specialist societies, such as the German Society of Anesthesiology and Intensive Care Medicine (DGAI). To improve EOL care in ICUs run by anesthesiologists in Germany, therapeutic indications have to be clean-cut, followed by decision making and implementation by the main players, the physicians and nurses, the patients, their legal representatives and families.

## Additional files

Additional file 1: Figure S1. EOL items Q12–15 of high implementation and high relevance (sufficient Category 1). Data are presented as “blob-o-grams” were the number of respondents in each category is represented by a circle whose area is proportional to the number. Importance (x-axis) and status of implementation (y-axis) are rated on modified Likert scales. Q = Question. C1 = sufficient Category 1. (JPEG 1380 kb)

Additional file 2: Figure S2. EOL items Q16–19 of sufficient Category 1. Data are presented as “blob-o-grams” were the number of respondents in each category is represented by a circle whose area is proportional to the number. Importance (x-axis) and status of implementation (y-axis) are rated on modified Likert scales. Q = Question. C1 = sufficient Category 1. (JPEG 1461 kb)

Additional file 3: Figure S3. EOL items Q20, 21, 24, 29 of sufficient Category 1. Data are presented as “blob-o-grams” were the number of respondents in each category is represented by a circle whose area is proportional to the number. Importance (x-axis) and status of implementation (y-axis) are rated on modified Likert scales. Q = Question. C1 = sufficient Category 1. (JPEG 1446 kb)

Additional file 4: Figure S4. EOL items Q30–33 of sufficient Category 1. Data are presented as “blob-o-grams” were the number of respondents in each category is represented by a circle whose area is proportional to the number. Importance (x-axis) and status of implementation (y-axis) are rated on modified Likert scales. Q = Question. C1 = sufficient Category 1. (JPEG 1482 kb)

Additional file 5: Figure S5. EOL items Q36, 38, 39, 42 of sufficient Category 1. Data are presented as “blob-o-grams” were the number of respondents in each category is represented by a circle whose area is proportional to the number. Importance (x-axis) and status of implementation (y-axis) are rated on modified Likert scales. Q = Question. C1 = sufficient Category 1. (JPEG 1335 kb)

Additional file 6: Figure S6. EOL items Q47–50 of sufficient Category 1. Data are presented as “blob-o-grams” were the number of respondents in each category is represented by a circle whose area is proportional to the number. Importance (x-axis) and status of implementation (y-axis) are rated on modified Likert scales. Q = Question. C1 = sufficient Category 1. (JPEG 1354 kb)

Additional file 7: Figure S7. EOL items Q1–3 of low implementation and low relevance (inessential Category 2). Data are presented as “blob-o-grams” were the number of respondents in each category is represented by a circle whose area is proportional to the number. Importance (x-axis) and status of implementation (y-axis) are rated on modified Likert scales. Q = Question. C2 = inessential Category 2. (JPEG 1203 kb)

Additional file 8: Figure S8. EOL items Q22, 23, 25, 26 of high importance that are rarely implemented but are considered to be highly relevant (unsatisfactory Category 3). Data are presented as “blob-o-grams” were the number of respondents in each category is represented by a circle whose area is proportional to the number. Importance (x-axis) and status of implementation (y-axis) are rated on modified Likert scales. Q = Question. C3 = unsatisfactory Category 3. (JPEG 1486 kb)

Additional file 9: Figure S9. EOL items Q27, 28, 34, 35 of unsatisfactory Category 3. Data are presented as “blob-o-grams” were the number of respondents in each category is represented by a circle whose area is proportional to the number. Importance (x-axis) and status of implementation (y-axis) are rated on modified Likert scales. Q = Question. C3 = unsatisfactory Category 3. (JPEG 1452 kb)

Additional file 10: Figure S10. EOL items Q37, 40, 41, 43 of unsatisfactory Category 3. Data are presented as “blob-o-grams” were the number of respondents in each category is represented by a circle whose area is proportional to the number. Importance (x-axis) and status of implementation (y-axis) are rated on modified Likert scales. Q = Question. C3 = unsatisfactory Category 3. (JPEG 1448 kb)

## Abbreviations

ABCDs: Attitudes behaviours compassion and dialogue
AHDC: advanced health care directives
AND: allow natural death
BDA: Association of German anesthesiologists
CEO’s: chief executive officer
CTC: Comfort terminal care
DGAI: German society of anesthesiology and intensive care medicine
DNE: Do not escalate
DNR: Do not resuscitate
DRG: diagnosis related groups
EOL: end-of-life
ICU: Intensive care units
n: number
n. a.: status of implementation given without estimation of importance
Q: question
RID: Re-evaluate indication and De-escalate
SAPS II: Simplified acute physiology score II
SD: standard deviation
SOFA: Sequential organ failure assessment
SOPs: standard operating procedures
