# Supplementary figures and images for: End-of-life perceptions among physicians in intensive care units managed by anesthesiologists in Germany: a survey about structure, current implementation and deficits
Source: BMC Anesthesiol. 2017 Jul 11;17:93. doi: 10.1186/s12871-017-0384-5 (PMC5504988; doi:10.1186/s12871-017-0384-5)

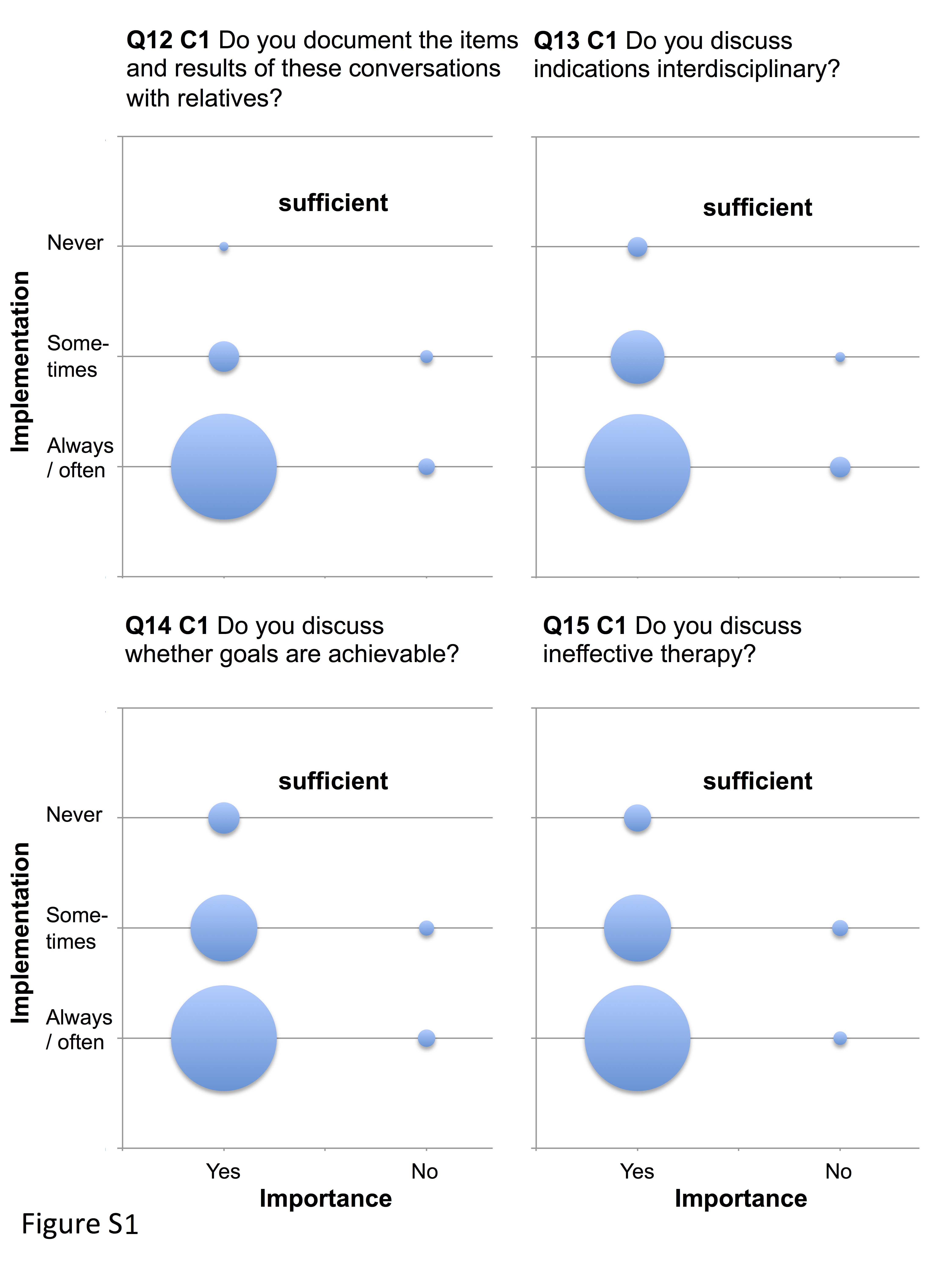

Supplement: Supplementary file 1 — EOL items Q12–15 of high implementation and high relevance (sufficient Category 1). Data are presented as “blob-o-grams” were the number of respondents in each category is represented by a circle whose area is proportional to the number. Importance (x-axis) and status of implementation (y-axis) are rated on modified Likert scales. Q = Question. C1 = sufficient Category 1. (JPEG 1380 kb) [file 12871_2017_384_MOESM1_ESM.jpg]

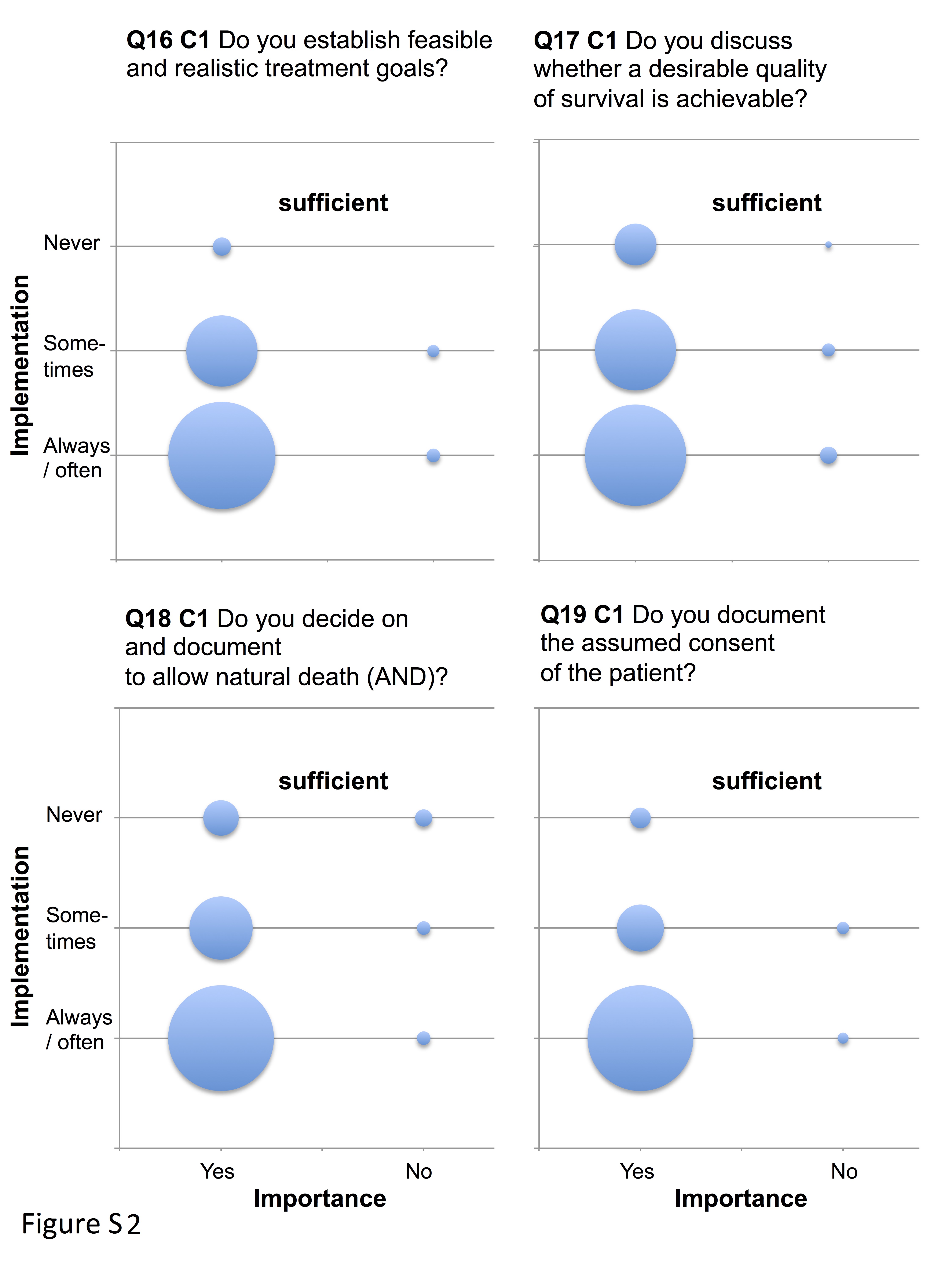

Supplement: Supplementary file 2 — EOL items Q16–19 of sufficient Category 1. Data are presented as “blob-o-grams” were the number of respondents in each category is represented by a circle whose area is proportional to the number. Importance (x-axis) and status of implementation (y-axis) are rated on modified Likert scales. Q = Question. C1 = sufficient Category 1. (JPEG 1461 kb) [file 12871_2017_384_MOESM2_ESM.jpg]

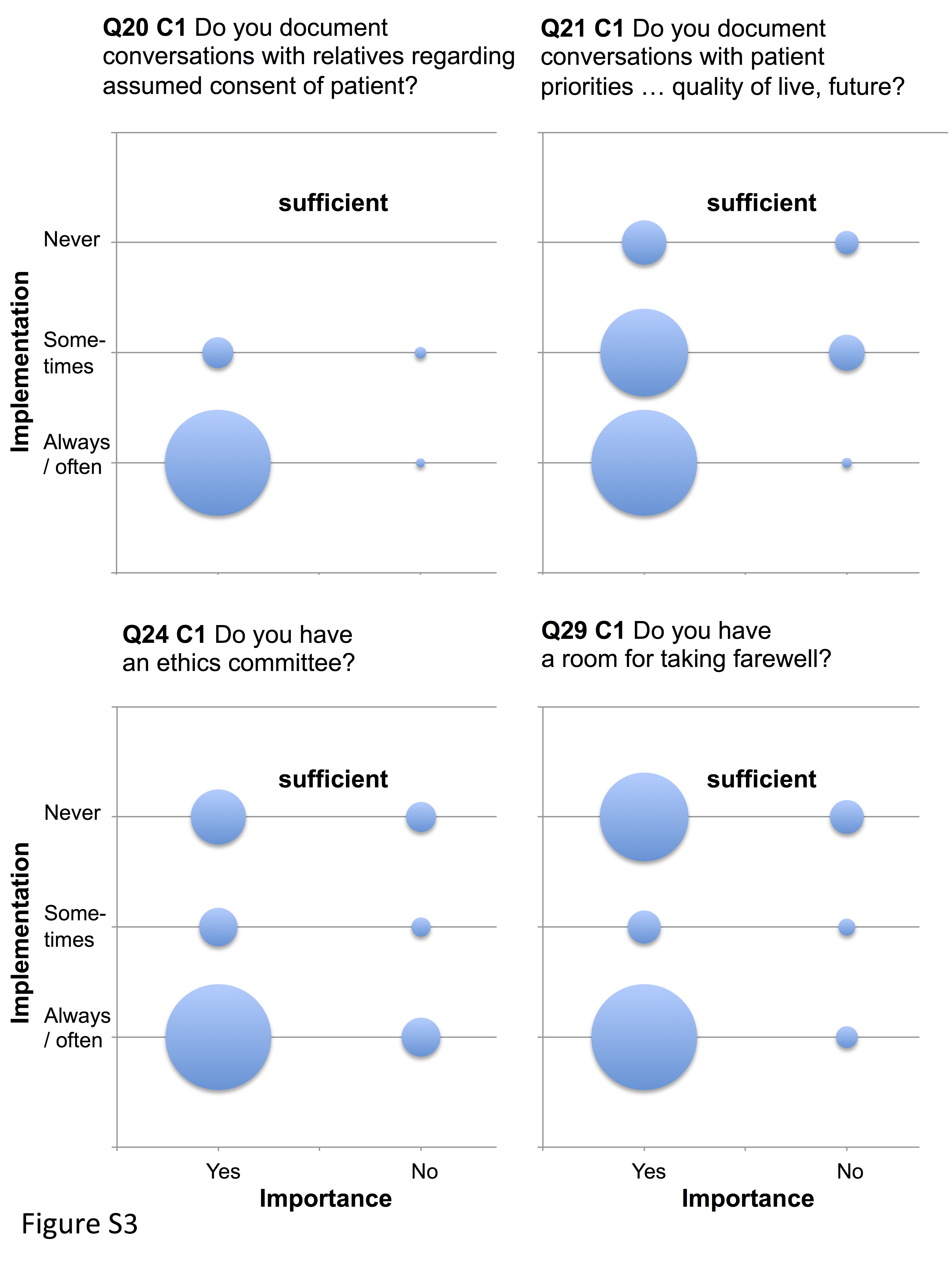

Supplement: Supplementary file 3 — EOL items Q20, 21, 24, 29 of sufficient Category 1. Data are presented as “blob-o-grams” were the number of respondents in each category is represented by a circle whose area is proportional to the number. Importance (x-axis) and status of implementation (y-axis) are rated on modified Likert scales. Q = Question. C1 = sufficient Category 1. (JPEG 1446 kb) [file 12871_2017_384_MOESM3_ESM.jpg]

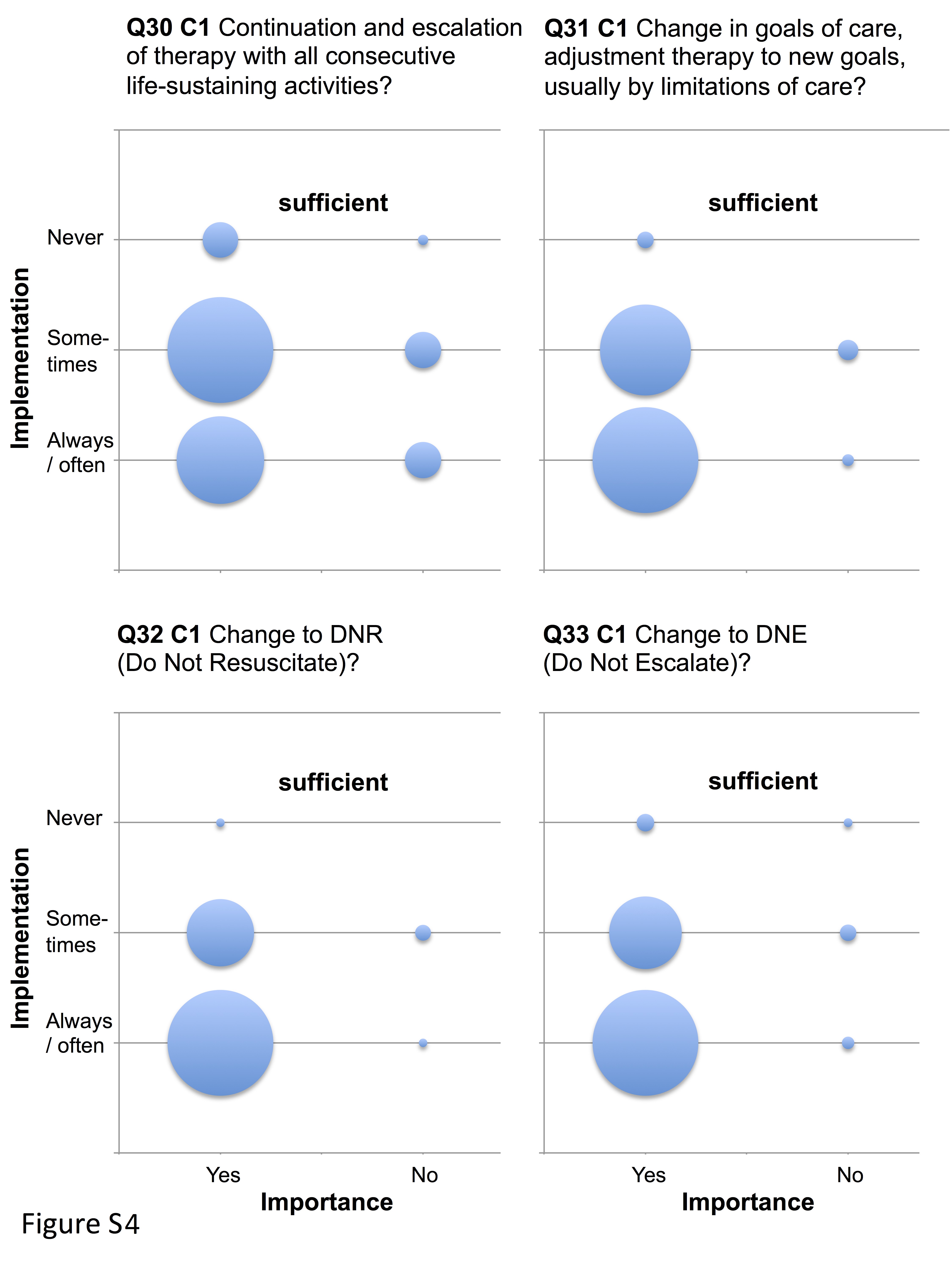

Supplement: Supplementary file 4 — EOL items Q30–33 of sufficient Category 1. Data are presented as “blob-o-grams” were the number of respondents in each category is represented by a circle whose area is proportional to the number. Importance (x-axis) and status of implementation (y-axis) are rated on modified Likert scales. Q = Question. C1 = sufficient Category 1. (JPEG 1482 kb) [file 12871_2017_384_MOESM4_ESM.jpg]

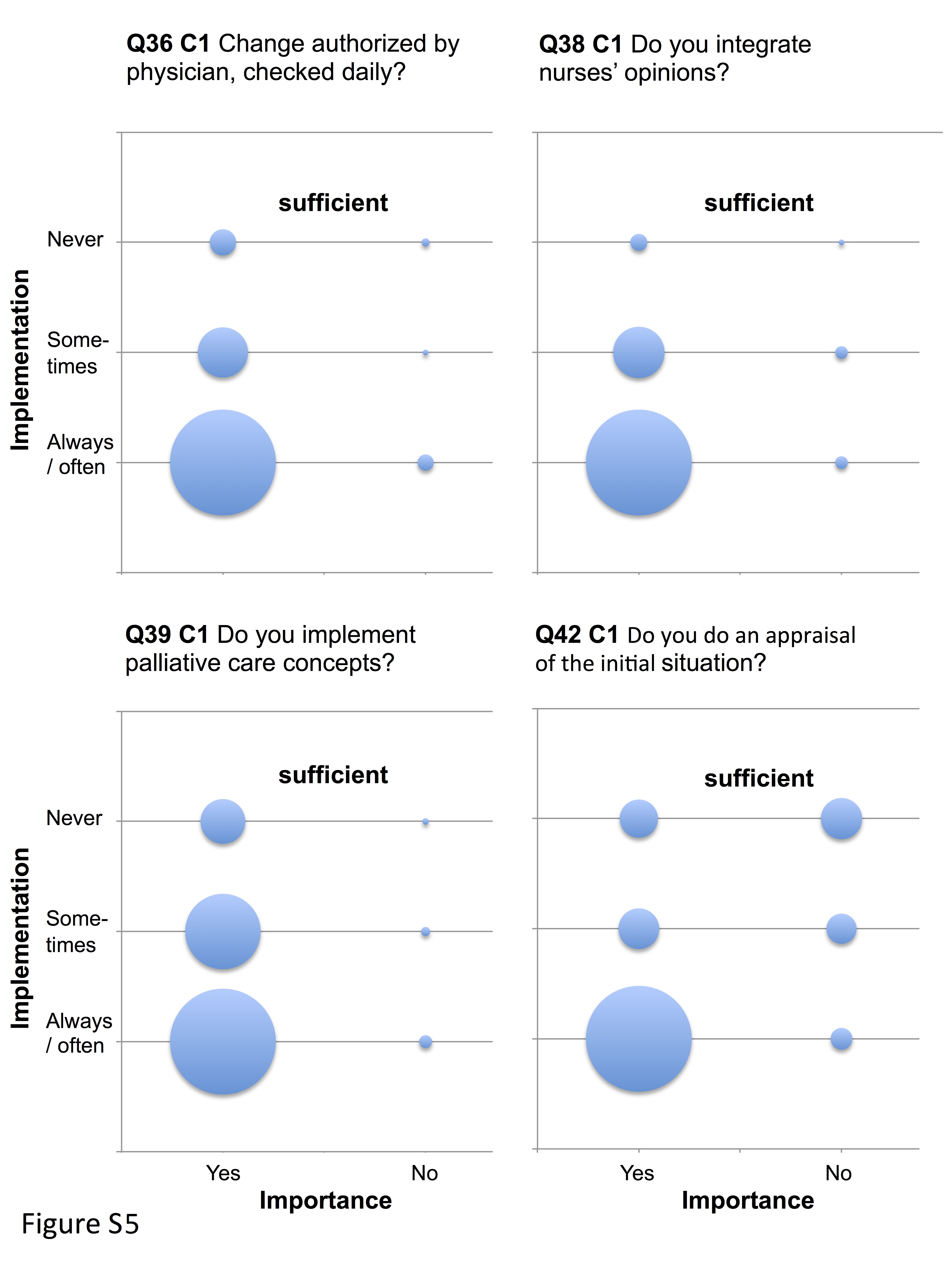

Supplement: Supplementary file 5 — EOL items Q36, 38, 39, 42 of sufficient Category 1. Data are presented as “blob-o-grams” were the number of respondents in each category is represented by a circle whose area is proportional to the number. Importance (x-axis) and status of implementation (y-axis) are rated on modified Likert scales. Q = Question. C1 = sufficient Category 1. (JPEG 1335 kb) [file 12871_2017_384_MOESM5_ESM.jpg]

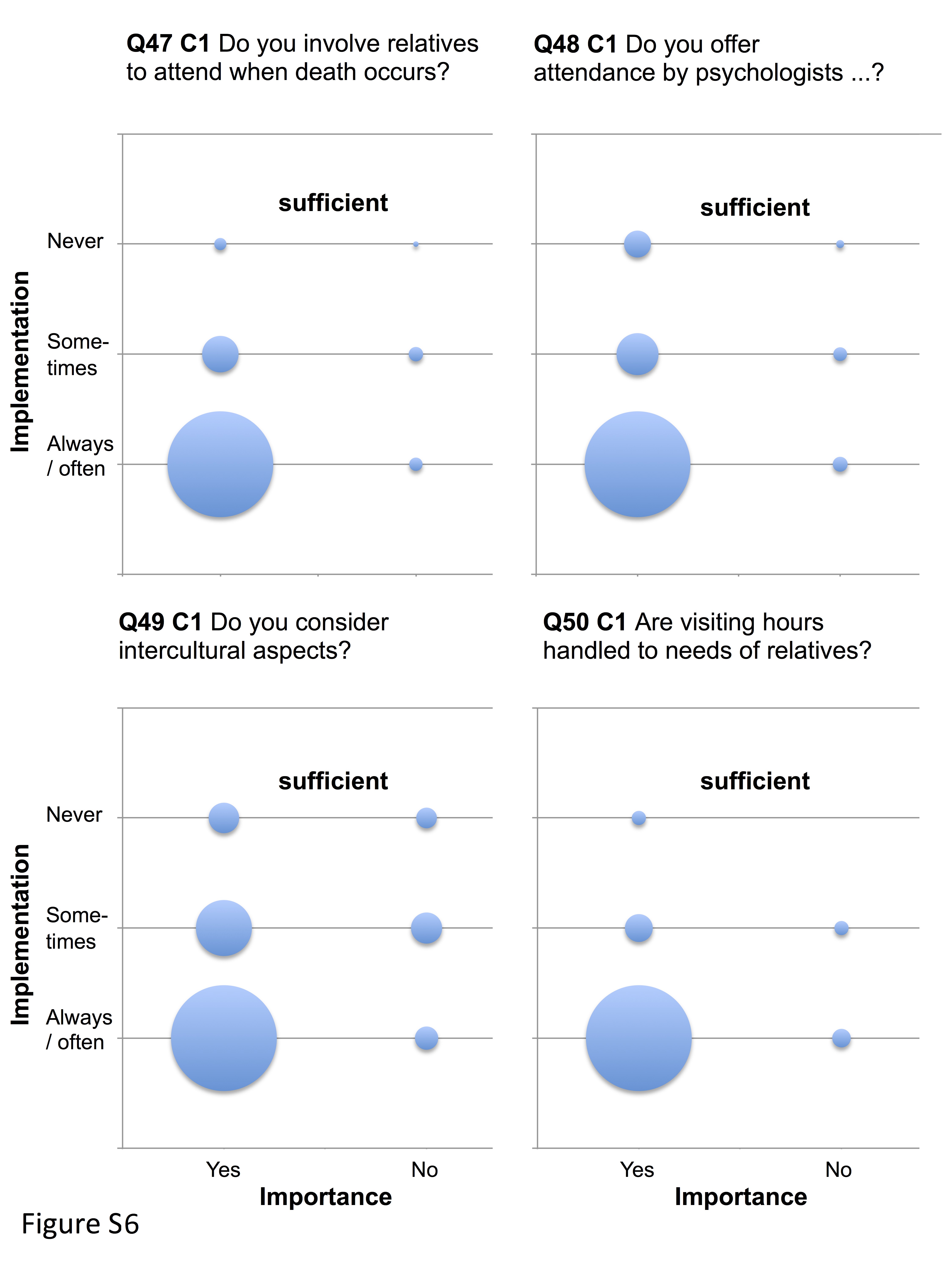

Supplement: Supplementary file 6 — EOL items Q47–50 of sufficient Category 1. Data are presented as “blob-o-grams” were the number of respondents in each category is represented by a circle whose area is proportional to the number. Importance (x-axis) and status of implementation (y-axis) are rated on modified Likert scales. Q = Question. C1 = sufficient Category 1. (JPEG 1354 kb) [file 12871_2017_384_MOESM6_ESM.jpg]

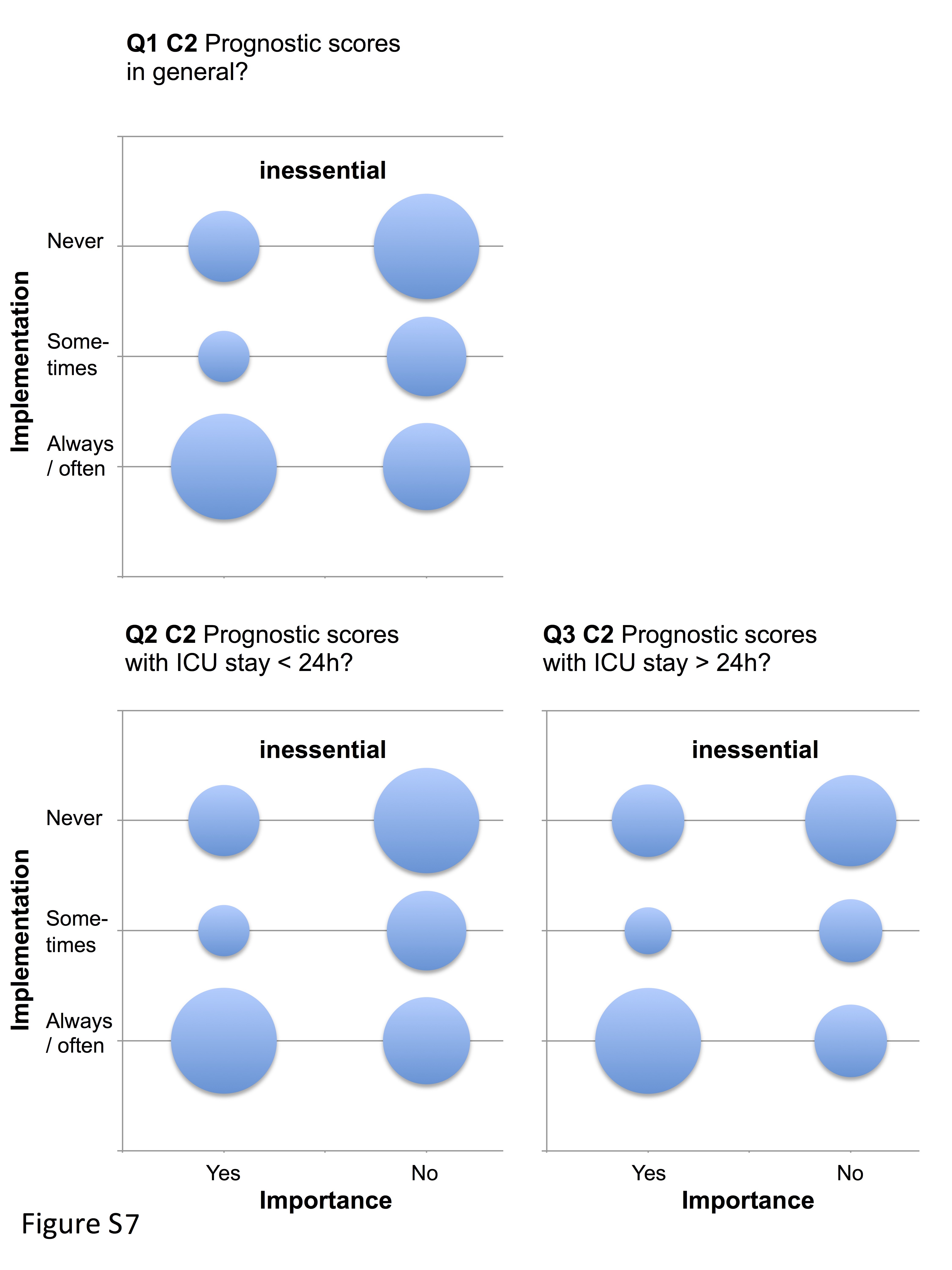

Supplement: Supplementary file 7 — EOL items Q1–3 of low implementation and low relevance (inessential Category 2). Data are presented as “blob-o-grams” were the number of respondents in each category is represented by a circle whose area is proportional to the number. Importance (x-axis) and status of implementation (y-axis) are rated on modified Likert scales. Q = Question. C2 = inessential Category 2. (JPEG 1203 kb) [file 12871_2017_384_MOESM7_ESM.jpg]

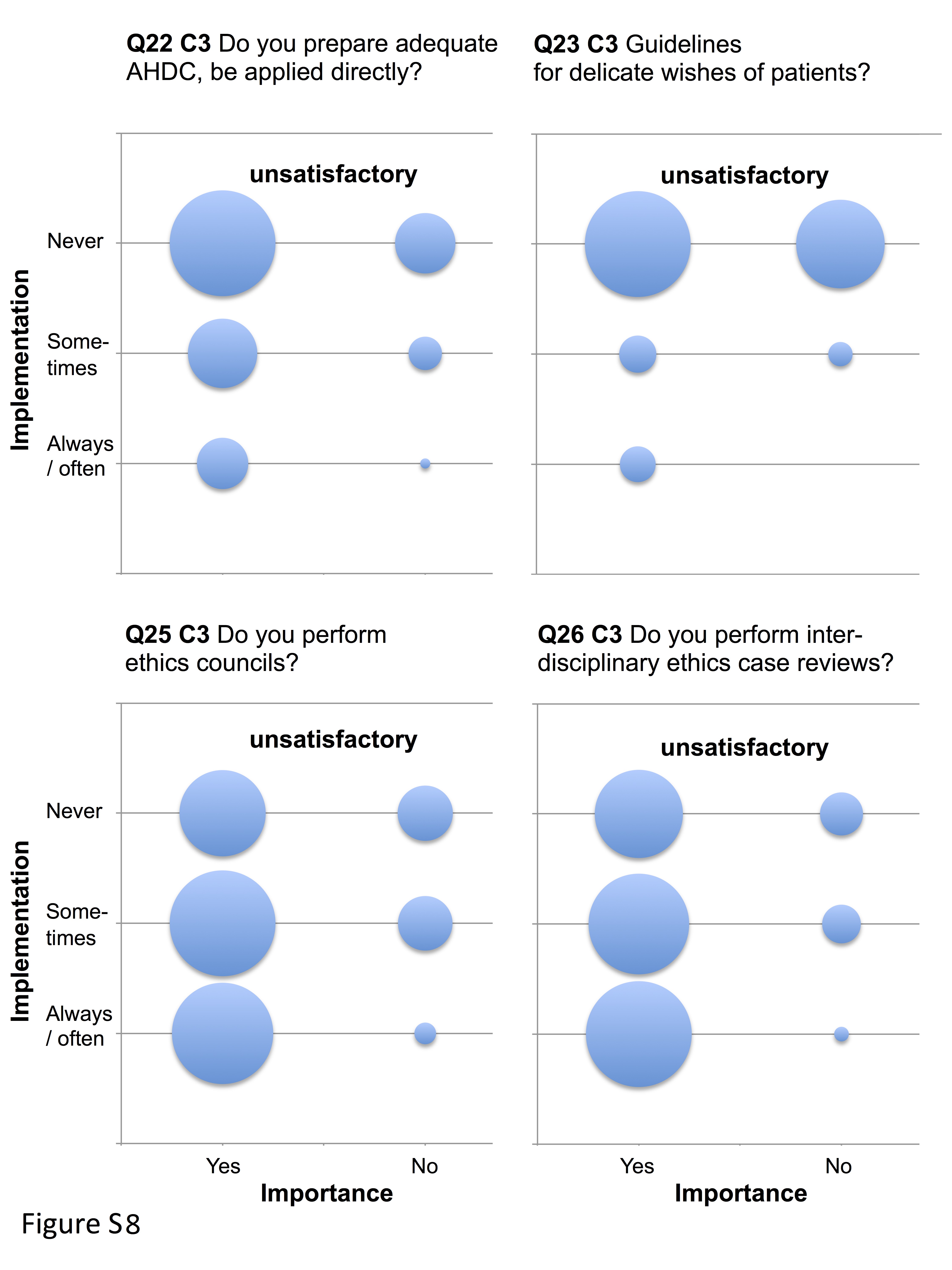

Supplement: Supplementary file 8 — EOL items Q22, 23, 25, 26 of high importance that are rarely implemented but are considered to be highly relevant (unsatisfactory Category 3). Data are presented as “blob-o-grams” were the number of respondents in each category is represented by a circle whose area is proportional to the number. Importance (x-axis) and status of implementation (y-axis) are rated on modified Likert scales. Q = Question. C3 = unsatisfactory Category 3. (JPEG 1486 kb) [file 12871_2017_384_MOESM8_ESM.jpg]

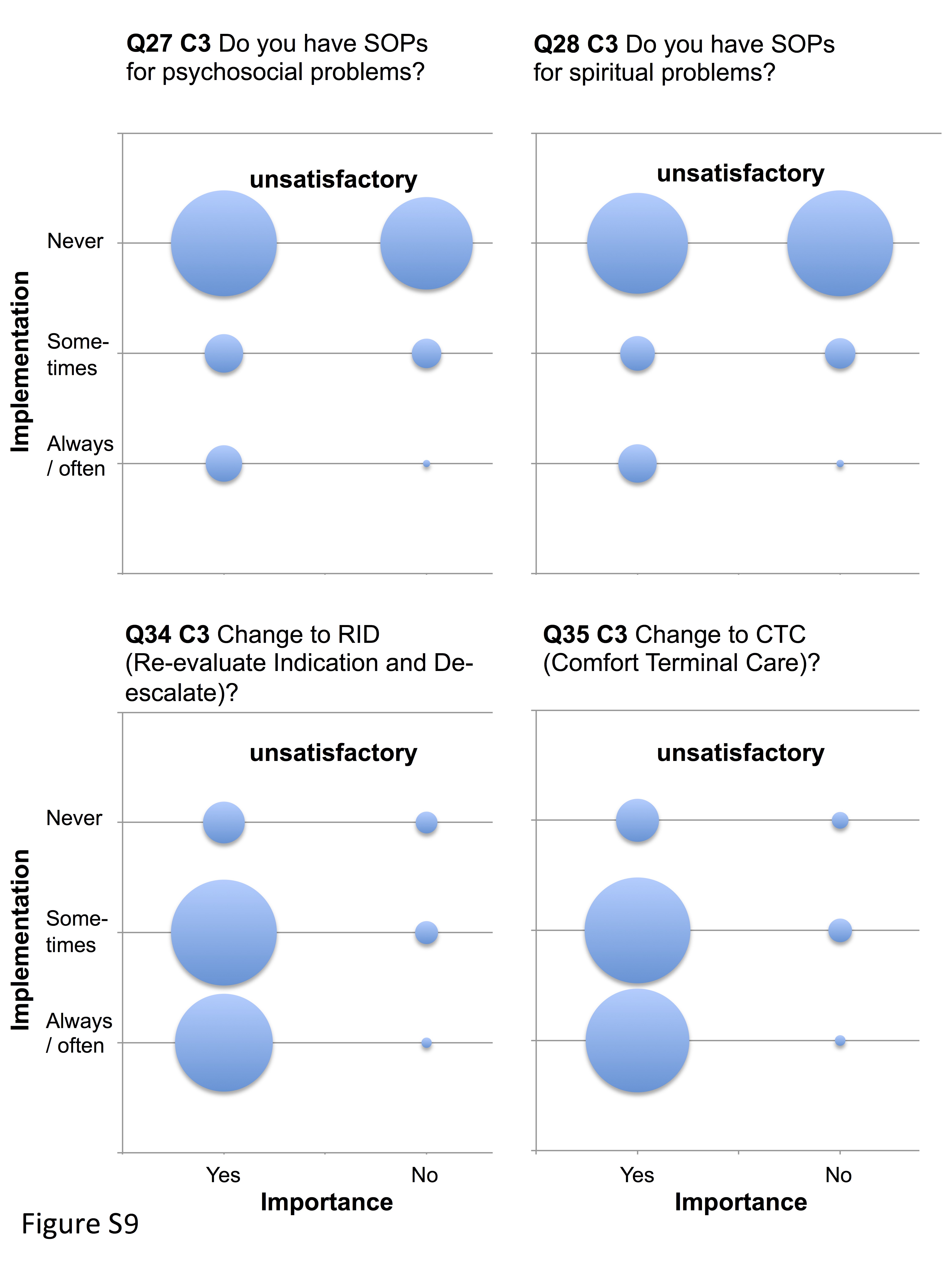

Supplement: Supplementary file 9 — EOL items Q27, 28, 34, 35 of unsatisfactory Category 3. Data are presented as “blob-o-grams” were the number of respondents in each category is represented by a circle whose area is proportional to the number. Importance (x-axis) and status of implementation (y-axis) are rated on modified Likert scales. Q = Question. C3 = unsatisfactory Category 3. (JPEG 1452 kb) [file 12871_2017_384_MOESM9_ESM.jpg]

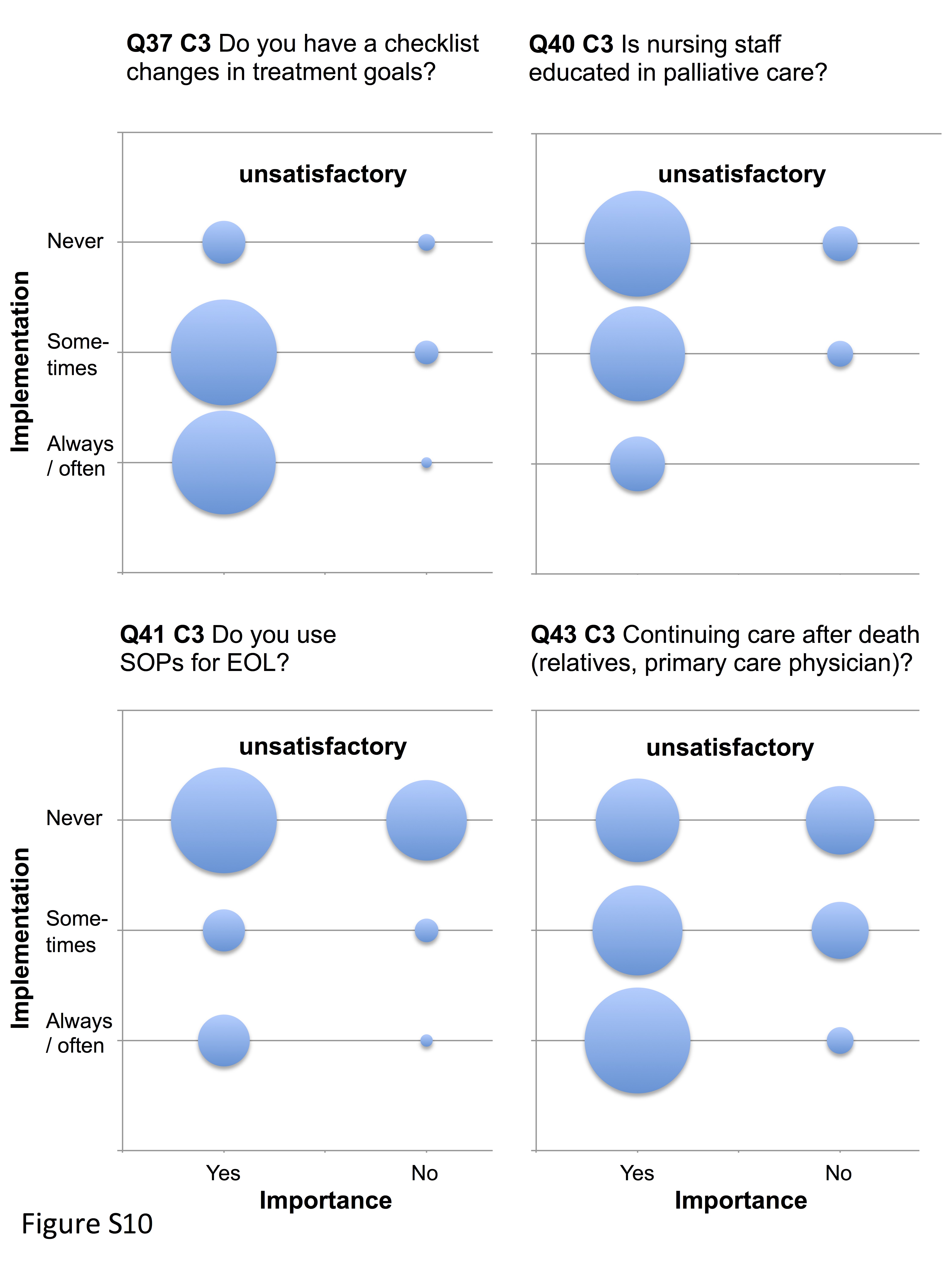

Supplement: Supplementary file 10 — EOL items Q37, 40, 41, 43 of unsatisfactory Category 3. Data are presented as “blob-o-grams” were the number of respondents in each category is represented by a circle whose area is proportional to the number. Importance (x-axis) and status of implementation (y-axis) are rated on modified Likert scales. Q = Question. C3 = unsatisfactory Category 3. (JPEG 1448 kb) [file 12871_2017_384_MOESM10_ESM.jpg]
